# The pennycress (*Thlaspi arvense* L.) nectary: structural and transcriptomic characterization

**DOI:** 10.1186/s12870-017-1146-8

**Published:** 2017-11-14

**Authors:** Jason B. Thomas, Marshall E. Hampton, Kevin M. Dorn, M. David Marks, Clay J. Carter

**Affiliations:** 1 Department of Plant & Microbial Biology, University of Minnesota Twin Cities, Saint Paul, MN 55108 USA; 2 Department of Mathematics & Statistics, University of Minnesota Duluth, Duluth, MN 55812 USA; 3 Department of Plant Pathology, Kansas State University, Manhattan, Kansas, 66506 USA

**Keywords:** Pennycress, Nectar, Nectaries, Nectary, *Thlaspi arvense*, Brassicaceae

## Abstract

**Background:**

Pennycress [*Thlaspi arvense* L (Brassicaceae)] is being domesticated as a renewable biodiesel feedstock that also provides crucial ecosystems services, including as a nutritional resource for pollinators. However, its flowers produce significantly less nectar than other crop relatives in the Brassicaceae. This study was undertaken to understand the basic biology of the pennycress nectary as an initial step toward the possibility of enhancing nectar output from its flowers.

**Results:**

Pennycress flowers contain four equivalent nectaries located extrastaminally at the base of the insertion sites of short and long stamens. Like other Brassicaceae, the nectaries have open stomates on their surface, which likely serve as the sites of nectar secretion. The nectaries produce four distinct nectar droplets that accumulate in concave structures at the base of each of the four petals. To understand the molecular biology of the pennycress nectary, RNA was isolated from ‘immature’ (pre-secretory) and ‘mature’ (secretory) nectaries and subjected to RNA-seq. Approximately 184 M paired-end reads (368 M total reads) were de novo assembled into a total of 16,074 independent contigs, which mapped to 12,335 unique genes in the pennycress genome. Nearly 3700 genes were found to be differentially expressed between immature and mature nectaries and subjected to gene ontology and metabolic pathway analyses. Lastly, in silico analyses identified 158 pennycress orthologs to Arabidopsis genes with known enriched expression in nectaries. These nectary-enriched expression patterns were verified for select pennycress loci by semi-quantitative RT-PCR.

**Conclusions:**

Pennycress nectaries are unique relative to those of other agriculturally important Brassicaceae, as they contain four equivalent nectaries that present their nectar in specialized cup-shaped structures at the base of the petals. In spite of these morphological differences, the genes underlying the regulation and production of nectar appear to be largely conserved between pennycress and *Arabidopsis thaliana*. These results provide a starting point for using forward and reverse genetics approaches to enhance nectar synthesis and secretion in pennycress.

**Electronic supplementary material:**

The online version of this article (10.1186/s12870-017-1146-8) contains supplementary material, which is available to authorized users.

## Background

With the concurrent growth in world population [1] and decline in biodiversity [2], efficient use of our land and energy resources is crucial. To partially address this issue, pennycress (*Thlaspi arvense*) is being developed as a renewable biodiesel feedstock that also provides vital ecosystems services [3]. Pennycress seeds have high oil content (20–36%, wt/wt), making it competitive with other oilseed crops, such as soybean and canola [3]. The oil from pennycress seeds can be processed to power diesel engines as well as commercial airplanes and jets [4].

Since pennycress is a winter annual with a short life cycle, it can be intercropped within corn and soybean rotations, adding biodiversity to our agroecosystems and utilizing the 16 million hectares of soil that normally lie fallow in the fall, winter, and spring in the Upper Midwest [3]. Thus, pennycress is a highly marketable ‘cash’ cover crop that can increase farmers’ profits while suppressing weeds [5], preventing nutrient leaching [6], and protecting the soil from erosion [7]. Farmers have generally been resistant to growing cover crops, citing limited profitability, weed problems from escaped progeny, and high management requirements [7]; however, current pennycress breeding programs are addressing these issues. Therefore, pennycress is projected to have widespread implementation on farms throughout the Upper Midwest because of its marketable uses and ecosystem services, as well as its cold hardiness relative to other existing cover crops, including canola and camelina.

Pennycress may provide yet another major ecosystem service by offering essential nutritional resources to pollinators in the form of nectar and pollen during a critical time of the year, early spring. The health of pollinator populations is crucial to food production because nearly 75% of global crop species depend on some level of animal-mediated pollination [8]. In 2010 pollination services were estimated to be worth $29 billion in the United States alone [9]. The importance of pollinators in food security has recently received attention from the White House, which noted pennycress as a potentially valuable pollinator resource that needs additional research [10].

Insects are readily attracted to pennycress flowers, even though they produce relatively low levels of nectar when compared to the closely related canola (*Brassica napus*) and camelina (*Camelina sativa*) [11]. It is unclear why pennycress attracts many diverse pollinators yet offers little nectar, but it is possible that the insects are instead feeding on its pollen. One interesting finding was that honey bees were a significant minority of visitors to pennycress flowers [11], possibly due to pennycress having relatively small flowers with low levels of nectar production. For example, pennycress flowers are approximately one-third the size of those from camelina, and produce roughly one-eighth of the total nectar sugar [11]. These results suggest that there may be opportunities to enhance pennycress floral traits in order to benefit honey bees and other pollinators. The identification and development of pennycress cultivars with increased nectar, pollen, and larger floral displays may benefit both wild pollinator and domesticated honey bee populations, which are in decline, while at the same time increasing crop yields [12]. In particular, extra nutritional resources would be highly valuable for honey bees in the early spring months, which is a critical time of the year for both managed hives and wild bees [13, 14].

The nectary is the gland responsible for producing the complex mixture of compounds found in nectars. Members of the Brassicaceae typically contain four nectaries per flower [15, 16]. In Arabidopsis and canola, two lateral nectaries secrete >99% of the total nectar while the two smaller median nectaries are much less productive [15, 16]. More vascular tissues subtend the lateral than median nectaries, which may be partially responsible for the disparate nectar secretion between these two organs (A.R. Davis in [17]).

We previously identified a large number of genes expressed in the nectaries of *Arabidopsis thaliana* and *Brassica rapa*, two species related to pennycress, via transcriptional profiling [18, 19]. Subsequent work demonstrated mechanistic roles for 19 of these genes in nectary function [20]. In particular, a pathway for the synthesis and secretion of nectar sugars in the Brassicaceae was discovered (described in detail in *Results* and *Discussion*) [21, 22].

Given the rapid development of pennycress as a cover and cash crop, and its likely widespread implementation on millions of acres in the upper Midwest, the study described in this manuscript was undertaken to understand the basic biology of the pennycress nectary as an initial step toward the possibility of enhancing nectar output from its flowers.

## Results

### Nectary structure and nectar presentation

**Fig. 1:**
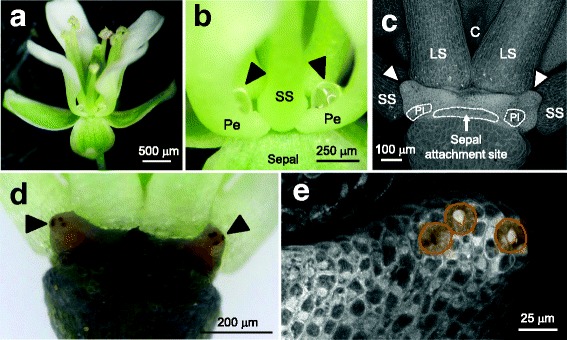
Pennycress nectary structure and nectar presentation. **a** Whole pennycress flower. **b** Two nectar droplets (arrowheads) cradled by petals on either side of a short stamen (SS); Pe = petal. **c** Two pennycress nectaries located at the base of long stamens, arrowheads point to tips of nectaries. LS = long stamen, C = carpel, PI = petal insertion site (outlined with dashed line). This image was created via confocal microscopy and a z-stack compilation of 12 images. **d** Nectaries (arrowheads) from a Stage 15 flower stained with iodine/potassium iodide. The darkly-staining punctate structures are stomates, which are the sites of nectar secretion. **e** Confocal image of nectary tip with stomates outlined

Pennycress flowers develop four equivalent nectaries, which are located extrastaminally at the base of the insertion sites in between short and long stamens (Fig. 1). Each nectary is located apically to a petal attachment site and produces a nectar droplet cradled by a cup-shaped structure at the petal base (Fig. 1a, b). Lastly, nectary ultrastructure was analyzed by starch staining and confocal microscopy. These analyses demonstrated that the nectary surface contains stomates (nectarostomata) that accumulate starch in actively secreting nectaries (Fig. 1d), and that these stomates appear to be open (Fig. 1e). The occluded material in the stomas shown in Fig. 1e is most likely cross-linked nectar solutes, although nectary stomatal occlusion has been previously reported [23].

### Transcriptional profiling of nectaries

**Fig. 2:**
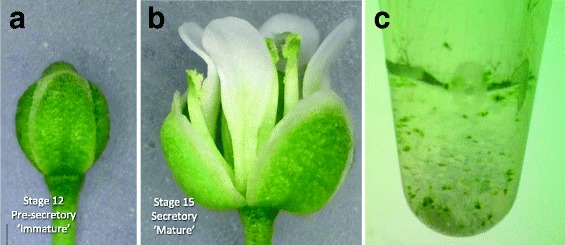
Floral stages from which nectary tissues were collected. **a** Immature and (**b**) mature flowers equivalent to Stage 12 and 15 from Arabidopsis, respectively. Stage 12 (immature) nectaries are pre-secretory, whereas Stage 15 (mature) nectaries produce nectar. **c** Example of one replicate of manually collected nectaries. A single replicate for RNA isolation consisted of ~200 pooled nectaries

Nectar production usually begins at anthesis in the Brassicaceae, which also holds true for pennycress. To understand the events that precede nectar production, and what machinery defines an actively secreting nectary, pennycress nectaries were manually collected at two developmental time points: pre-secretory (‘immature,’ equivalent of Stage 11–12 in Arabidopsis; Fig. 2) and secretory (‘mature,’ equivalent of Stage 14–15 in Arabidopsis; stages defined in [24]). Total RNA was subsequently isolated from both mature and immature nectaries in triplicate and processed for Illumina Hi-Seq 2500 sequencing (including assessment of RNA quality, polyA purification, and library creation). Approximately 184 M paired-end 50 bp reads (368 M total reads) were obtained from the equivalent of a single sequencing lane, which were subsequently trimmed, assembled, and mapped to the pennycress genome. In summary, 16,074 independent contigs were generated (Additional file 1), which mapped to 12,335 unique pennycress genes (Additional file 2). The number of total reads and the nearest Arabidopsis gene (as identified via BLASTP) can be found for all contigs in Additional file 1. As multiple contigs often mapped to the same pennycress locus (due to incomplete coverage, alternative splicing, etc.), reads for such contigs were subsequently summed for each pennycress locus in Additional file 2. Interestingly, 376 contigs did not map to loci in the draft pennycress genome, but 242 of these had significant identity to Arabidopsis loci (Additional file 1).

### Differential expression of genes between immature and mature nectaries

**Fig. 3:**
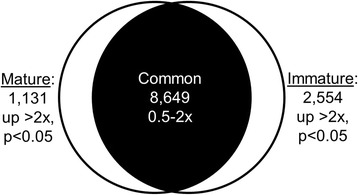
Venn diagram of numbers of co- and differentially expressed genes between immature and mature nectaries. RNA-seq reads were assembled into contigs de novo and mapped to loci in the draft pennycress genome [30]. Data summarized from Additional file 6

Nearly 3700 pennycress genes were found to have >2-fold average difference in expression between immature and mature nectaries (*p* < 0.05), with 2554 and 1131 being higher in immature and mature nectaries, respectively (Fig. 3). Since these genes demark the differences between a nectary preparing for nectar secretion and one actively secreting nectar, these loci were subjected to gene ontology and pathway analyses via the ‘Statistical Overrepresentation Test’ from the Panther Classification System (http://pantherdb.org/) using the nearest Arabidopsis orthologs as the input (Additional file 3). Loci upregulated in immature versus mature nectaries include 270 genes involved in ‘biosynthetic processes’ (overrepresented, *p* = 1.96 × 10^−24^), particularly ones involved in fatty acid biosynthesis (17 genes, *p* = 1.97 × 10^−4^) and translation (157 genes, *p* = 1.13 × 10^−36^). Conversely, mature nectaries preferentially expressed genes over-representing processes involved in vesicular-mediated transport (41 genes, *p* = 9.26 × 10^−3^) and ubiquitin-protein ligase activity (30 genes, *p* = 1.26 × 10^−2^), among others (Additional file 3). Not surprisingly, many genes displayed similar differential expression between immature and mature nectaries in both pennycress and Arabidopsis (Additional file 4).

### Genes preferentially expressed within nectary tissues

**Fig. 4:**
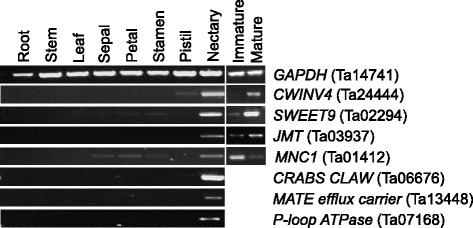
Identification and validation of pennycress genes with enriched expression in nectaries. Putative pennycress orthologs of Arabidopsis genes with known nectary-enriched expression were identified by BLASTP against translated contigs derived from RNA-seq. Semi-quantitative RT-PCR was used to verify these orthologs as shown in the left panel. The right panel was used to validate differential expression of several of these genes in immature (Stage 12) versus mature (Stage 15) flowers as identified by RNA-seq in this study. The pennycress gene number and associated Arabidopsis orthologs are as follows: CWINV4 (Ta24444; AT2G36190), SWEET9 (Ta02294; AT2G39060), JMT (Ta03937; AT1G19640), MNC1 (Ta01412; AT1G74820), CRABS CLAW (Ta06676; AT1G69180), MATE efflux carrier (Ta13448; AT1G23300), P-loop ATPase (Ta07168; AT5G60760), GAPDH (Ta14741; AT3G04120)

A previous study using microarray analyses identified 270 Arabidopsis loci that displayed >3-fold expression in nectaries over each of 13 other reference tissues (e.g. leaf, petal, stem, root, etc.) [19]. These Arabidopsis loci were compared to the genes expressed in pennycress nectaries (those from Additional file 2) to gauge the extent to which the processes involved in nectar secretion may be conserved. Nearly 60% (158 out of 270) of the Arabidopsis loci displaying nectary-enriched expression were represented by the top respective pennycress ortholog (as determined by BLASTP) in the pennycress nectary transcriptome (Additional file 5). The enriched expression in nectaries for several of these putative orthologs was subsequently confirmed by semi-quantitative PCR (Fig. 4). It is possible that orthologs to the remaining nectary-enriched Arabidopsis genes (112 out of 270) not identified in the pennycress transcriptome may still be expressed but not identified in this analysis due to the presence of closely related paralogs in both genomes.

### Conservation of a pathway for nectar secretion

Our prior work discovered a pathway for the synthesis and secretion of nectar sugars in the Brassicaceae and Solanaceae, and suggested conservation elsewhere [21, 22]. In our working model nectar is formed by pre-nectar sugars and other metabolites derived from phloem [21, 22]. The pre-nectar may travel symplastically through several layers of parenchyma via plasmodesmata where it is deposited as starch in the amyloplasts of immature flowers [21, 22]. At anthesis (flower opening) the starch is degraded and sucrose is re-synthesized from those sugars by sucrose phosphate synthases and/or sucrose synthases. These sucrose molecules are then exported into the extracellular space by the plasma membrane-localized sucrose transporter SWEET9 [21]. At the extracellular space CELL WALL INVERTASE4 (CWINV4) cleaves the sucrose into the hexose monomers fructose and glucose [22]. The hexoses create a negative water potential causing water to move towards the sugars, thereby forming nectar droplets [22].

Putative pennycress orthologs to each of the genes, or representative enzymes involved in each of the nectar producing steps described above, were identified in the RNA-seq data. Moreover, not only were these genes represented in the pennycress transcriptome, they were highly expressed (read counts in top 1.5% of all genes expressed in mature nectaries) and significantly upregulated (*p* < 0.05) in mature over immature nectaries (Additional file 2, Figs. 4 and 5). These genes encode proteins likely involved in the steps of starch degradation (β-amylase 1, BAM1, Ta10910), sucrose synthesis (sucrose synthase, SUS, Ta03482), sucrose export (SWEET9, Ta02294), and extracellular hydrolysis (CWINV4, Ta24444).

**Fig. 5:**
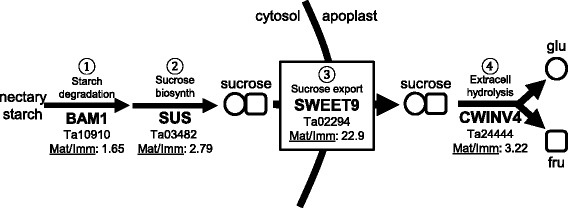
Pennycress genes likely involved in nectar synthesis and secretion. The primary steps of nectar production in Arabidopsis are ① starch degradation, ② sucrose biosynthesis, ③ sucrose export via SWEET9, and ④ extracellular hydrolysis by CELL WALL INVERTASE4. Putative pennycress orthologs to these genes, or representative enzymes involved in each of these steps, were identified from RNA-seq-derived contigs. The gene abbreviation, gene number, and expression ratio between mature and immature nectaries are listed (Mat/Imm). BAM, β-amylase; SUS, sucrose synthase. Each of these loci were significantly (*p* < 0.05) more highly expressed in mature over immature nectaries

## Discussion

Most Brassicaceae flowers, including those of *Brassica* spp. and Arabidopsis, contain two sets of non-equivalent nectaries. Lateral nectaries produce the vast majority of nectar and are located at the base of short stamens, whereas median nectaries produce little or no nectar and are found that the base of long stamens [16]. Interestingly, pennycress nectary structure appears to be unique, at least relative to other agriculturally important Brassicaceae, in that they have four equivalent nectaries that secrete nectar droplets into concave structures at the base of petals (Fig. 1). The two nectaries located on the same medial face of each flower are contiguous with one another via a ridge of tissue (Fig. 1c), whereas nectaries on the same lateral face are separated by a short stamen (Fig. 1d); it is unclear if the tissue linking two contiguous nectaries plays a functional role in nectar secretion, such as common links to the supplying vasculature. Pennycress’ small, open-type flowers with easily accessible nectar droplets suggest that it is visited by generalist pollinators, which is indeed what has been recently reported [11, 25]. The primary visitors to pennycress flowers include small bees, flies, and butterflies [11, 25].

Nectaries from some species have stomates on their surfaces that strongly stain for starch in actively secreting flowers, including Arabidopsis and *Brassica rapa* [26]. This also holds true for pennycress, which generally have ~3–5 stomates per nectary (e.g. Fig. 1d, e). It has been suggested that nectary stomates are ‘modified’ and permanently open [27], but it appears this phenomenon has only been thoroughly tested in a single species [23, 28, 29].

Transcriptional profiling of pennycress nectaries was conducted at two developmental timepoints to obtain a snapshot of which genes define a nectary preparing for nectar production and one in the midst of active secretion. RNA-seq-derived contigs (Additional file 1) mapped to over 12,000 pennycress genes (Additional file 2), which is equivalent to 45% of the predicted genes present in the draft pennycress genome [30]. This suggests that mRNAs present in nectaries were sampled to near exhaustion.

Comparative expression analyses of immature and mature nectaries shed light on potentially novel discoveries relative to the process of nectar production not noted before. In particular, genes involved in lipid and protein synthesis were highly upregulated in immature nectaries (Additional files 3, 4 and 6), whereas vesicular trafficking and ubiquitination processes were enriched in mature nectaries. Nectar production is a secretory process, which is at least partly dependent on vesicular fusion with the plasma membrane to release some nectar components [26]. Thus it is highly probable that immature nectaries actively synthesize lipids that are needed to produce membranous materials that in turn will be used for merocrine- or granulocrinte-type (vesicle-based) secretion in mature nectaries.

The pennycress nectary transcriptome was also compared to that of Arabidopsis. The fact that the direct pennycress ortholog to ~60% of Arabidopsis genes with highly enriched expression in nectaries were also identified as being expressed in pennycress nectaries provides compelling evidence that the mechanisms involved in nectary maturation and nectar secretion are conserved between the two species (Additional file 4; Additional file 5). Moreover, genes likely involved in the full pathway of nectar sugar synthesis and secretion are significantly upregulated in mature pennycress nectaries (Fig. 5). Interestingly, sucrose-phosphate synthases are not highly expressed in pennycress nectaries, even though they are required for nectar production in Arabidopsis [21]. However, an ortholog to Arabidopsis *SUCROSE SYNTHASE1* (*SUS1*, Ta03482) is highly expressed in mature pennycress nectaries (Fig. 5). While sucrose synthases are usually thought to be involved in sucrose catabolism [31], in some cases they are involved in sucrose synthesis [32]. Thus, it may be that Ta03482 functionally replaces the role of sucrose-phosphate synthases in mature pennycress nectaries. It should also be noted that the high level of CWINV4 expression (Figs. 4 and 5) is consistent with the finding that pennycress nectar is hexose-dominant, containing little or no sucrose [11].

## Conclusions

This study demonstrated that pennycress nectary morphology is relatively novel in the Brassicaceae in having four functionally equivalent nectaries. Yet, the presence of nectarostomata, and transcriptional profiling of pennycress nectaries, strongly suggests that the mechanisms of nectar production are largely maintained in this family. This study provides an initial step toward potentially enhancing nectar production in pennycress. For example, several genes and pathways are known positive [21, 22, 24, 33–35] and negative [36, 37] regulators of nectar production in other species. These loci could serve as targets to enhance nectar output in pennycress. Future studies should also address nectary ultrastructure in more detail (e.g., subtending vasculature), as well as nectar composition and nectar secretion dynamics.

## Methods

### Plant material and growth conditions

*Thlaspi arvense* cultivar MN108 plants were grown in 4″ pots with Sun Gro LC8 soil supplemented with Osmocote Flower and Vegetable Smart-Release Plant Food (14–14-14; according to the manufacturer’s instructions) under a 16 h day/8 h night cycle, photosynthetic photon flux of 200 μmol m^−2^ s^−1^, and a temperature of 22 °C.

### Nectary sample preparation and RNA isolation

Two types of RNA samples, were prepared from pennycress nectaries: mature nectaries (equivalent to Stage 14–15 flowers from *Arabidopsis thaliana*; stages defined in [17, 38]), and immature nectaries (equivalent to Stage 11–12 flowers from *Arabidopsis thaliana*). Mature nectaries secrete nectar, whereas immature nectaries are pre-secretory. All nectary tissues were dissected by hand from the flowers of primary inflorescences of ca. 40 day-old plants. Due to the small size of nectaries, dissections took place over several days from 4 to 8 h after dawn (h.a.d.), which are the peak hours of nectar secretion in related Brassicaceae [19]. Isolated nectaries were pooled in RNAlater™ solution (Ambion, Austin, TX) on ice, and stored at 4 °C prior to RNA extraction. Up to four nectaries were collected per flower, with approximately 200 nectaries being processed as a single RNA sample. Each biological replicate was represented by nectaries pooled from different sets of plants.

### RNA extraction, library creation, and sequencing

RNA was extracted from nectaries by mechanical disruption with a microcentrifuge pestle, and using the RNAqueous®-Micro RNA isolation kit (Ambion, Austin, TX) with Plant RNA Isolation Aid (Ambion, Austin, TX). Agarose gel electrophoresis and UV spectrophotometry were used to assess RNA quality of all samples prior to submission to the University of Minnesota Genomics Center for barcoded library creation and Illumina HiSeq 2500 sequencing. Six TruSeq RNA v2 libraries were created from ~500 ng of total RNA (triplicate samples for immature and mature nectaries) and sequenced via 50 bp, paired-end runs on the HiSeq 2500 using Rapid chemistry. All libraries were pooled and sequenced across two lanes to achieve the equivalent of one lane output. This generated ~368 M total reads (~184 M paired-end reads) and the average quality scores were above Q30.

### RNA-seq quality, assembly, normalization, and statistical analyses

The sequenced reads from immature and mature nectary samples were assembled separately using Trinity [39], which automatically takes read quality and consistency into account, and then merged with the transcriptome of Dorn et al. [40] to yield 49,933 contigs. Reads were mapped to this contig set by NCBI’s blastn, with an E-value cutoff of 0.00001, and the counts upper-quartile normalized. Normalized counts were fitted to a negative binomial distribution using DESeq v1.6.1 [41]. The resulting *p*-values from DESeq were filtered by restricting to contigs with a 50% or greater change in mean expression between immature and mature samples, and with at least 100 or more normalized counts in either sample type. The Benjamini-Hochberg method was used to control the false discovery rate of contigs determined to be differentially expressed to 0.05 [42].

### RT PCR validation

Total RNA from various pennycress tissues was isolated as described for nectaries above and cDNA was created with the Promega Reverse Transcription System (A3500, Promega Corp., Madison, WI, USA) according to the manufacturer’s instructions. Negative controls without reverse transcription were also used to ensure there was no contaminating genomic DNA. Semi-quantitative RT PCR reactions consisted of 7 μL H_2_O, 2.5 μL gene reverse primer (10 μM), 2.5 μL gene forward primer (10 μM), 12.5 μL GoTaq® (Promega Corp., Madison, WI, USA), and 0.5 μL cDNA from immature or mature flowers. GAPDH was used as a positive control, as it displayed relatively low variation of expression across tissues. Target genes were selected based on presumed orthology to Arabidopsis genes with known enriched expression in nectaries (see [19]). The sequences of all oligonucleotide primers used in this study are available in Additional File 7. Samples were held at initially at 95 °C for 120 s, followed by a repeating cycles of 95 °C for 30s, 55 °C for 30s, and 72 °C for 70s. 5.5 μL of DNA were removed from reactions after 27, 30, 35 and 40 cycles. Before each removal, cycles were held at 72° for 5 min and then cooled to 21 °C. 5 μL of PCR product was imaged on 1% agarose/TAE gel using SYBR Safe (S33102, ThermoFisher) for visualization.

### Microscopy

To image nectary starch, fresh flowers, with sepals and petals removed, were stained in Lugol’s Solution (L6146, Sigma Chemical) for ~30 s, rinsed in water for ~3 min, and immediately imaged with a stereomicroscope.

To examine nectary morphology via confocal microscopy a method modified from Landis et al. was used [43]. Briefly, fresh flowers were fixed in glutaraldehyde fixing solution (4% glutaraldehyde in 25 mM NaPO_4_, pH 7.3) overnight at 4 °C. Floral tissues then went through a series of ethanol/water rinses: 50% for 90 min at −20 °C, 70% for 90 min at −20 °C, 85% for 60 min at 4 °C, 95% for 60 min at 4 °C, and 95% overnight at 4 °C. Tissues were then rinsed in 100% ethanol for 1 h, again in 100% ethanol overnight at 4 °C, and again in 100% ethanol for 1-2 h at room temperature. Histo-Clear (National Diagnostics) was used to remove the waxy cuticle covering the nectary by a series of Histo-Clear:ethanol rinses at room temperature: (1:0, 3:1, 1:1, 1:3, 0:1) for 1 h each. Sequentially diluted ethanol rinses were then used to rehydrate the tissues (75%, 50%, 25%, 0%), each for 1 h. To prepare samples for fluorescence microscopy, flowers were stained in 1% (*w*/*v*) aniline blue mixed with phosphate buffered saline, vacuum infiltrated for 10 min, incubated overnight, and rinsed in water prior to imaging. A Nikon A1 Spectral Confocal Microscope at the University of Minnesota Imaging Center was used to image whole stained flowers excited at 405 nm with emission collected from 500 to 550 nm. 3-dimensional images were generated from z-stack series with NIS- Elements software.

### Functional group overrepresentation analysis in immature versus mature nectaries

Genes displaying >2-fold and statistically significant differences in expression between mature and immature nectaries (e.g. Additional file 6, as determined by DESeq and FDR via the Benjamini-Hochberg method described above, *p* < 0.05) were analyzed using the ‘Statistical Overrepresentation Test’ tool via the PANTHER Classification System using the default settings and Bonferroni correction for multiple testing (*p* < 0.05) ([44] http://pantherdb.org/).

## Additional files

Additional file 1: Pennycress nectary contigs and reads. Full list of all nectary contigs, associated pennycress loci, top Arabidopsis hit, and normalized read counts. (XLS 5381 kb)

Additional file 2: Pennycress nectary reads by locus. Summed reads for all pennycress loci represented by transcripts in the nectary transcriptome. (XLSX 2041 kb)

Additional file 3: GO Slim analyses**.** Biological processes, molecular functions, and cell components over- or underrepresented in genes differentially expressed between immature and mature nectaries. (XLSX 130 kb)

Additional file 4: Pennycress and Arabidopsis differential expression. List of genes commonly differentially regulated between immature and mature nectaries in pennycress and Arabidopsis. (XLSX 200 kb)

Additional file 5: Nectary-enriched genes. Pennycress orthologs to Arabidopsis genes with known enriched expression in nectaries. (XLSX 44 kb)

Additional file 6: Differentially expressed genes. Full list of genes displaying >2-fold difference and *p* < 0.05 in expression between immature (Stage 11–12) and mature (Stage 14–15) nectaries. (XLSX 607 kb)

Additional file 7: Oligonucleotides used in this study. List of primers used for RT PCR validation experiments. (XLSX 19 kb)

## Abbreviations

BAM: β-amylase
CWINV: Cell wall invertase
RT PCR: Reverse transcription polymerase chain reaction
SUS: Sucrose synthase
